# A policy Delphi study to validate the key implications of data sharing (KIDS) framework for pediatric genomics in Canada

**DOI:** 10.1186/s12910-021-00635-1

**Published:** 2021-06-09

**Authors:** Vasiliki Rahimzadeh, Gillian Bartlett, Bartha Maria Knoppers

**Affiliations:** 1 Stanford Center for Biomedical Ethics, Stanford University, 1215 Welch Rd, Stanford, CA 94305 USA; 2 School of Medicine, University of Missouri, 7 Hospital Drive Suite MA306N Medical Sciences Bldg, Columbia, MO 65211 USA; 3 Centre of Genomics and Policy, McGill University, 740 Ave Docteur Penfield, Suite 5200, Montreal, QC H3A0G1 Canada

**Keywords:** Genomics, Data sharing, Pediatrics, Data protection, Ethics review, Delphi

## Abstract

**Background:**

The highly sensitive nature of genomic and associated clinical data, coupled with the consent-related vulnerabilities of children together accentuate ethical, legal and social issues (ELSI) concerning data sharing. The Key Implications of Data Sharing (KIDS) framework was therefore developed to address a need for institutional guidance on genomic data governance but has yet to be validated among data sharing practitioners in practice settings. This study qualitatively explored areas of consensus and dissensus of the KIDS Framework from the perspectives of Canadian clinician-scientists, genomic researchers, IRB members, and pediatric ethicists.

**Methods:**

Twelve panelists participated in a three-round online policy Delphi to determine the *desirability*, *feasibility*, *relative importance* and *confidence* of twelve individual statements of the KIDS Framework. Mean and IQR were calculated from panelists’ ratings to determine the strength of consensus and polarity. Qualitative content analysis of panelists’ written responses was used to assess degree of support. Statements were validated when their combined ratings and qualitative rationales indicated high-moderate consensus (at least 70% agreement across two contiguous categories), low to no polarity (IQR at least 1.0) and strong support.

**Results:**

Nine original, and one new statement reached consensus. These statements outlined essential elements of the informed consent process, including a realistic evaluation of benefits and risks and assurance of future ethics oversight for secondary data use. Discrepant views on appropriate protections for anonymized and coded i.e. de-identified genomic data were primary sources of dissensus.

**Conclusions:**

The validated statements provide institutions with empirically supported best practices for sharing genomic and associated clinical data involving children from the perspectives of key stakeholders. Concerted efforts to quantify informational risks that can be conveyed to patients and families are further needed to align data sharing policy with stakeholder priorities.

**Supplementary Information:**

The online version contains supplementary material available at 10.1186/s12910-021-00635-1.

## Background

Large-scale databases e.g., OMIM, ClinGen and Orphanet that contain patient pheno-and genotypic-level data have accelerated advances in understanding gene-disease correlations [1]. Next generation sequencing and access to quality, annotated data aid clinicians in molecular diagnosis of individuals with Mendelian and other diseases with suspected genetic etiology. The availability of data assets necessarily relies on procuring them from patient sources either during the course of clinical care or participation in research. Because many genetic diseases present early in life, patient sources include undiagnosed children for whom both a scientific and ethical need justifies access, use and exchange of their genomic data [2, 3]. Although studies demonstrate that whole genome/exome sequencing can increase diagnostic yield by as much as 25% [4], interoperable sharing of children’s genomic data across clinical and research environments remains a key bottleneck in the discovery pipleline for rare genetic disease [5].

Children’s consent-related vulnerability, coupled with the highly identifying nature of genomic data can accentuate ethical, legal and social issues (ELSI) of sharing such data [6]. As computational capacities evolve to support more sophisticated geno/phenotypic analyses, so too must oversight bodies such as institutional review boards (IRB) [7, 8] and data access committees (DAC) be responsible for ensuring data protections are commensurate with anticipated benefits and realistic informational risks to children and their families [9, 10]. Recent studies suggest there is considerable debate on how data sharing benefits and risks should be interpreted, contextualized and communicated to prospective data contributors generally [10], and when involving children specifically [11, 12].

To address a need for improved ethics guidance, the lead author developed the Key Implications for Data Sharing (KIDS) framework for pediatric genomics in consultation with expert members of a special pediatric taskforce within the Global Alliance for Genomics and Health [13]. While the KIDS Framework lays a principled foundation for the responsible sharing of genomic and associated clinical data involving children, its potential implementation has yet to be evaluated empirically.

This article presents empirical results from an online policy Delphi study that combined quantitative and qualitative data collection to address the following research question: *How do Canadian pediatricians, genomic researchers, ethicists and bioethical scholars evaluate the ethical-legal, social and scientific factors of genomic and associated clinical data sharing involving children?* It furthermore explored areas of consensus and polarization in the ELSI debate surrounding responsible data sharing practices involving children.

## Methods

### Study design

A mixed methods policy Delphi design was used to evaluate 12 individual policy statements of the KIDS Framework from the perspectives of Canadian pediatricians, genomic researchers, IRB chairs and pediatric bioethicists. First proposed by Turoff in 1970, the policy Delphi is an “organized method for correlating views and information pertaining to a specific policy area and for allowing the respondents representing such views and information the opportunity to react to and assess differing viewpoints” [14]. Through a controlled input and feedback process using closed surveys, select experts in a Delphi panel (i) formulate the policy issue, (ii) explore policy options, (iii) determine initial positions, (iv) evaluate underlying reasons for these positions, and finally (v) re-evaluate policy options. The policy issue was first formulated, and initial policy options determined (phases i-iii) based on an earlier systematic review of the literature and consensus committee workshop [15]. Phases iv-v are reported in this paper.

### Panel selection

Delphi panelists represent individuals with diverse and well-informed perspectives on the policy issue of interest [15]. A purposive sampling strategy was used to identify prospective panelists who were both professionally and geographically representative of pediatric genomic stakeholders in Canada [16]. Electronic mail invitations were sent to panelists who were involved in any one of six pediatric genomic project teams funded by Genome Canada between 2012 and 2017, and met at least one of following professional criteria:

- Clinician-scientist.
- Genomic researcher.
- Member of the Institutional Review Board that granted project approval to the funded project.
- Pediatric ethicist.
- ELSI researcher..

### Data collection

All Delphi survey data were collected online using LimeSurvey between January and October 2018. Panelists were allotted 2 weeks to complete each Delphi Round, and a follow up email reminder was sent on the 3rd week. Panelists also remained anonymous to each other throughout the study to ensure confidentiality, enable independent rating and reduce undue influences namely between junior and more senior panelists. Panelists could also modify their answers at any time using a unique survey token. An informational landing page preceded each survey round whereby panelists agreed to participate prior to launching the online survey to ensure an ongoing consent process. Between three and five project-naïve individuals piloted each round of surveys for comprehension, format and clarity prior to distribution. The first author managed data collection and analysis. The authors have combined training in genomic science, biomedical ethics—with a special focus on issues of genetics/genomics involving pediatric populations in Canada—as well as knowledge translation. Many panelists had previous collaborated on, or were familiar with the authors’ work in these areas, enhancing participant trust in the research team.

### Data analysis

A classification system according to consensus ranking, polarity and support was used to assess ratings for 12 individual statements from the KIDS Framework. Panelists used a 4-point Likert scale to rate each statement on two of four categories—relative importance, feasibility, desirability or confidence—with 1 indicating most, and 4 indicating least important, feasibile, desirable and confident [17]. Mean and inter-quartile range were calculated from Likert ratings to determine the (i) degree of consensus and (ii) strength of polarity. Recommended thresholds for consensus—high, moderate, and low—and polarity—strong, weak and none—were established from the published Delphi literature [18] and are provided in Additional file 1. This quantitative data was triangulated with written, qualitative responses Delphi panelists were required to provide to justify their ratings. The supplemental rationales were then analyzed using content analysis [19, 20]. The lead author developed an initial codebook to analyze the qualitative data for each round, met routinely with co-authors to discuss results and coding from the prior round, and determined a consensus coding schema for the upcoming round.

Descriptive statistics [14] together with content analyses of rationales were used to determined “whether the group supported, opposed, or was ambivalent towards an option; whether the group was split…or whether no clear picture of support emerged” [18] for statements outlined in the KIDS Framework. Statements were validated if the combined ratings and qualitative responses indicated high consensus, low to no polarity and at least weak support. Panelists accessed a summary of how their ratings and responses from the previous round compared to the group. Statements indicating low consensus, high polarity or strong- to weak opposition were retained for re-rating and review in the subsequent round. All descriptive statistics and line-by-line qualitative coding were conducted in Microsoft Excel behind a password-protected server. Every survey concluded with the option to submit open-ended feedback on amending, eliminating or adding new statements.

## Results

40 invitations were sent to prospective panelists who met the above inclusion criteria. Ten panelists from the top four Canadian provinces for pediatric genomics research by gross federal funding participated in Round 1 (6 male, 4 female); 12 panelists in Round 2 (7 male, 5 female); and 12 panelists in Round 3 (6 male, 6 female)^1^ corresponding to response rates of 25%, 30 and 25%, respectively. Every round included at least one expert in pediatric medical genetics, genomics research, and research ethics (Table 1).

**Table 1:** Demographic characteristics of Delphi panel

Panel members	No. of panelists		
	Round 1 (n = 10)	Round 2 (n = 12)	Round 3 (n = 12*)
Gender			
Male	6 (60%)	7	6
Female	4 (40%)	5	6
Province			
Quebec	2 (20%)	4	4
Ontario	4 (40%)	4	4
British Columbia	2 (20%)	2	2
Nova Scotia	1 (10%)	1	1
Alberta	1 (10%)	1	1
Profession			
Clinician scientist	6 (60%)	7	6
ELSI researcher	2 (20%)	2	3
IRB Chair/member	2 (20%)	2	2
Pediatric ethicist	1 (10%)	1	1

*While 12 panelists participated in Round 3, only complete survey data from 10 panelists were reported in the analysis

### Round 1

Six statements met conditions for high consensus and low to no polarity in Round 1 (Table 2). Statements #1 and #3 resulted in the lowest combined ratings across both categories, indicating the strongest support for their validation in the overall framework as demonstrated by the excerpt below:

**Table 2:** Summary of results after panel ratings from Rounds 1 and 2 of the policy Delphi

#	KIDS Framework statement (13)	Measure^a^	Round 1 (n = 10)			Round 2 (n = 12)			Validated?
			Average	Consensus	Polarity	Average	Consensus	Polarity	
1	The best interests of children are primary	RI	1.4	High	None (0.488)	–	–	–	✔
		F	2	High	None (0.444)	–	–	–	
2	Children should be listened to, and involved in decision-making processes related to genomic and associated clinical data sharing in developmentally appropriate ways	D	2	High	None (0.222)	–	–	–	✔
		F	2.3	High	None (0.233)	–	–	–	
3	Parents should be informed in a transparent manner how their child’s genomic and associated clinical data will be securely managed and used	RI	1.2	High	None (0.177)	–	–	–	✔
		C	2.1	High	None (0.322)	–	–	–	
4	In a research context, data sharing infrastructures should enable children to withdraw consent to continued sharing of their genomic and associated clinical data when possible upon reaching the age of majority	D	1.8	High	None (0.177)	–	–	–	✔
		F	2.6	High	None (0.488)	–	–	–	
5	Parental authorization for ongoing, or future unspecified research should include the provision of information related to existing data governance	RI	1.6	High	None (0.711)	1.5	High	None (0.45)	✔
		D	1.7	High	Weak (0.9)	1.33	High	None (0.24)	
6	Values conveyed by family, legal guardians or primary care givers should be respected when possible	RI	1.7	High	None (0.677)	1.58	High	None (0.27)	✔
		F	2.6	Low	Strong (1.155)	2.5	High	None (0.45)	
7	Professionals involved in consent processes related to data sharing and data-intensive research have the responsibility to balance potential benefits and risks. A trained designate should be available to discuss these with parents at the time of consent	D	1.8	High	Weak (1.06)	1.5	High	None (0.45)	✔
		F	2.4	Low	None (0.5)	2.08	Mod	None (0.81)	
8	The decision to share pediatric genomic and associated clinical data should be supported by an evaluation of realistic risks and benefits	F	1.5	High	None (0.5)	–	–	–	✔
		C	1.7	High	None (0.455)	–	–	–	
9	Duplicative collection of genomic research data involving pediatric patients should be avoided	D	1.5	High	None (0.5)	–	–	–	✔
		F	2.4	High	None (0.488)	–	–	–	
10	Anonymized pediatric data should be made available via publicly accessible databases	D	2	High	Strong (1.11)	2.17	Low	Strong (1.42)	
		F	2	High	None (0.66)	1.92	Mod	None (0.81)	
11	Identifiable pediatric genomic and associated clinical data should be coded and made available through a controlled access process	D	1.6	High	Strong (1.115)	1.75	Mod	Strong (1.48)	
		F	1.8	High	Weak (0.844)	2	Mod	Weak (0.91)	
12	Providing children and their parents the opportunity to share genomic and associated clinical data is an obligation of those who generate such data	D	2	High	Strong (1.11)	1.67	Mod	Strong (1.15)	
		F	2.3	Low	Strong (1.122)	2.5	Low	Strong (1.18)	
13	Incidental (secondary) findings of clinically actionable genomic results should be made available	D	–	–	–	1.66	High	None (0.45)	✔
		F	–	–	–	2.33	High	None (0.7)	

^a^RI, relative importance; F, feasibility; C, confidence; D, desirability

This is the basic principle in paediatric medical ethics. Everything else depends on this—PQnd4.

In the open commentary, one panelist recommended adding the following statement: *Incidental (secondary) findings of clinically actionable, validated genomic results should be made available*. Six statements were retained for re-rating in Round 2 (#5–7, #10–12), as well as requests for amendments to four statements (#7, #10–12). The new Statement (#13) regarding return of incidental findings was also put to a panel vote during Round 2.

### Round 2

Panelists reached consensus on two of four retained Statements from Round 1; one new Statement (#13) and one amended Statement (#7). Table 2 presents the composite ratings from both Rounds 1 and 2. An inter-round comparison of the degree and direction of change between Rounds 1 and 2 were also calculated and are presented in Additional file 2. New ratings in Round 2 decreased on average 2.2 points, indicating heightened valuations of relative importance, feasibility and desirability after panelists reviewed results from Round 1. An amendment to Statement #7, as well as the addition of Statement #13 were both approved based on simple majority (Table 3). Statement #7 achieved the greatest degree of change according to the inter-round comparison, dropping 3 total Likert points. Panelists reasoned that a trained designate familiar with the anticipated benefits and risks of data sharing should be specified. A delegate should have patient-facing contact who can contextualize the anticipated risks and prospect of benefit to ensure a robust consent process. For example, “It is true that all individuals need to balance risks and benefits, but many involved in the process [of sharing genomic data] will not have the opportunity to actual [sic] address those issues with those giving consent to participation”. One panelist suggested that a genetic counselor, specifically, should be the designate. Another panelists objected to the need for a trained designate on the basis that all professionals involved in data sharing should be knowledable of the risks and benefits irrespective of whether they directly consent patients and their families,

**Table 3:** Voting results on amendments to 4 position statements after Round 2

#	Amended statement	Accept (n = 12)	Reject (n = 12)
7	Professionals involved in consent processes related to data sharing and data-intensive research have the responsibility to balance potential benefits and risks. *A trained designate should be available to discuss these with parents at the time of consent*	9 (75%)	3 (25%)
10	Anonymized pediatric data should be made available via *publicly accessible* databases	6 (50%)	6 (50%)
11	Identifiable pediatric genomic and associated data should be coded and made available through a controlled or registered access process	6 (50%)	6 (50%)
12	Providing children and their families the opportunity to share their genomic and associated data is an obligation of *researchers*	5 (42%)	7 (58%)
13	*Incidental (secondary) findings of clinically actionable, validated genomic results should be made available*	8 (67%)	4 (33%)

Amendments are indicated in italics

Whomever obtains consent from the participant/family, e.g. RA, coordinator etc., must however be able to sufficiently answer any questions they might have and be well enough informed to understand themselves the risks and benefits of data sharing—Pa7KH.

Both statistical and content analysis of the rationales for amendments to Statements #10–12 yielded mixed results (Table 3). Panelists who advocated for eliminating Statement #10, for example, argued that “true anonymization is a myth” (Pgw85), the informational risks associated with re-identification of data derived from genetic testing are too high “even with today’s technology” (8OYZ) and IRBs requires additional resources to ensure adequate data protection. One panelist underscored the fact that “Children are vulnerable and typically won’t be making this decision for themselves” to justify why public access to anonymized data should be discouraged. In contrast, one proponent justified their support for preserving Statement #10 by arguing that there should not be a separate risk threshold for children and adults:

I do not believe the risks to children in collecting and making available data (with protections considered adequate for adults) are unreasonable or that the level of risk alone would justify restricting its use. What is at issue probably stems more from a concern about the developing autonomy of children and the worry that choices might be made regarding data sharing that would not reflect the future adult’s preferences. However, we permit use of anonymized data without consent (sometimes even without REB review) for adults. I don’t think we can base this argument on level of risk (in the data sharing context) when we permit such practices for adults—Pa7KH.

Invoking the same respect for persons principle used to argue why Statement 10 should be eliminated, another panelist advocated for its preservation:

Denying families the ability to help other children (and possibly their own child as well) by sharing genomic and associated clinical data is paternalistic and can be seen as violating their autonomy.

Votes to amend Statement #11 were evenly split (Table 3), and panelists proposed three different access approaches for sharing coded genomic data i.e. de-identified data with a source key. Of those panelists who disagreed with both the original and amended Statement #11, most believed that recommending controlled access to coded data was premature given “the risks have not been well enough established.” Two panelists argued in favor of more stringent user authentication for controlled access to coded data, while another panelist suggested registered access was both a more efficient and economically viable option insofar as it ensures “robustness of the applicant authentication process”. The majority of panelists agreed that controlled access conferred an appropriate degree of security for coded genomic data, and contended that stricter access controls are counterproductive: “this is about not overprotecting children and delaying research” (PSbg4).

The amendment to Statement #12—proposing that only researchers are obliged to provide children and their families with opportunities to share data—likewise failed by a narrow margin (58%). Based on qualitative content analysis of the rationales against this amendment, panelists were conflicted on the perceived burden this obligation placed on clinicians, and that sharing “may not be appropriate for [sic] all types of genomic research.” Most panelists with a clinical background agreed that narrowing the obligations would be a missed opportunity:

Absolutely not!!!!! My clinical practice is consumed by doing genomic testing on patients, as is that of many of my pediatric specialty colleagues. Restricting data sharing to the narrow silo of researchers is going to miss vast amounts of highly valuable information, directly relevant to clinical practice. (PAw34)

Lastly, several panelists rejected in principle to any obligation to provide data sharing opportunities “even if highly desirable” (PHJR6).

### Round 3

**Fig. 1:**
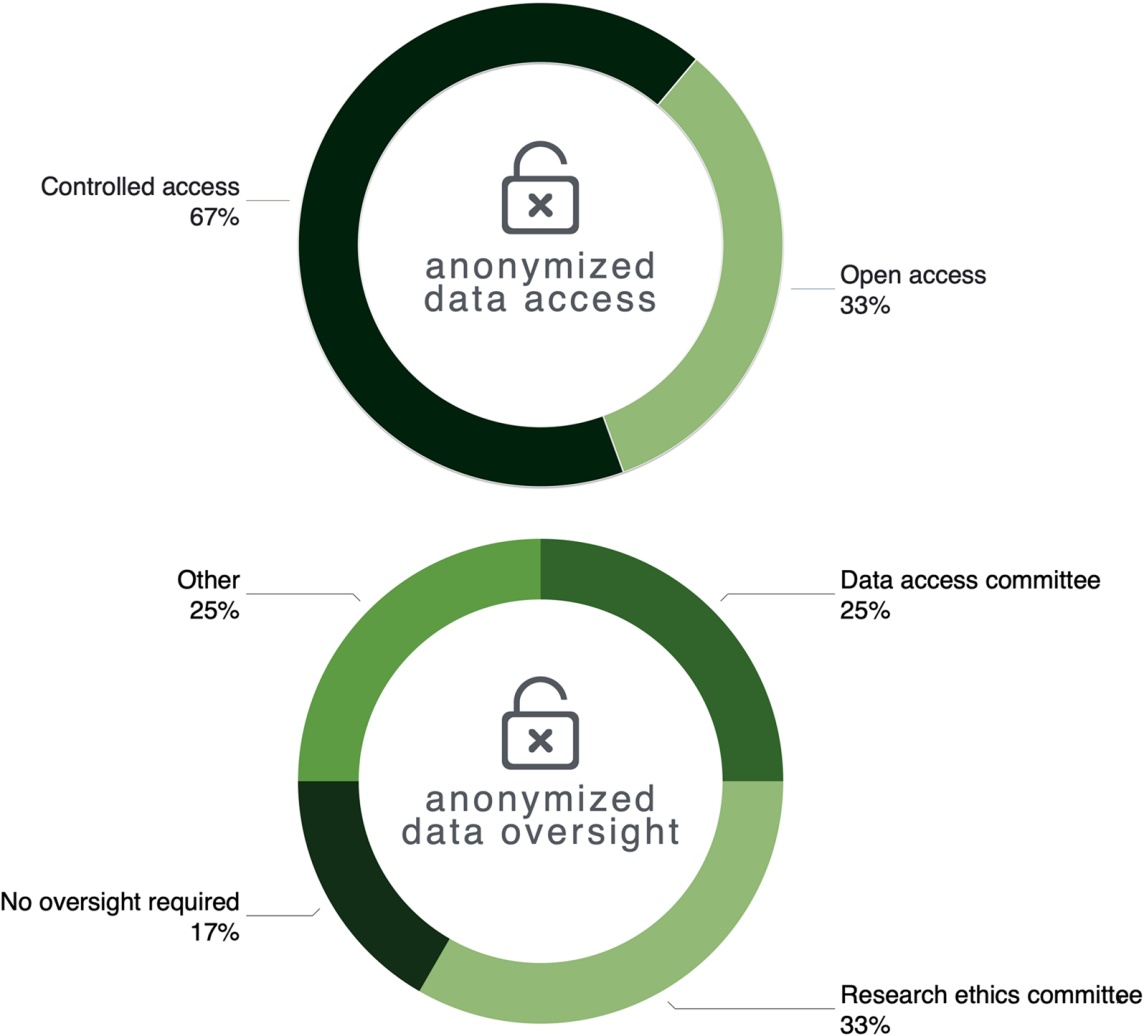
Results from Round 3 survey in response to the question, What is the most appropriate mechanisms of (i) data access and (ii) oversight for the responsible sharing of anonymized genomic data? N = 10*. *While 12 panelists in total participated in Round 3, only complete survey data from 10 panelists are reported

Relationships between appropriate data access and oversight for three Statements retained after Round 2 (#10–12) were explored in Round 3. A majority of panelists (67%) reported that controlled access was the most appropriate mechanism for sharing anonymized genomic data and named IRBs as the primary oversight bodies responsible for permitting such access (Fig. 1):

The concept of irrevocable anonymization needs to be recognized as a mythology–the genomic data itself contains the means to identify individuals. As such, free and open access put children at risk–PgWH5.

Excellent arguments can be made to demonstrate that modern genomic databases contain data of such detail that true anonymization is impossible–individuals can be identified with remarkable fidelity—PW85N.

Three of twelve panelists participating in Round 3, however, strongly disagreed with the above rationales and considered the “benefits [of sharing anonymized genomic data] outweigh the harms” (P3ew99). Furthermore, these panelists reasoned that the risks of sharing even anonymized genomic data via open access depended on the number of reported cases or variants available in a database where the data were shared and its provenance i.e. how the data was sourced:

If you have tons of children and just a list of variants, and maybe one additional source of data, then yes you may have more likelihood of knowing who that patient is. But this does not matter in the long-term if you ask patients and their families especially in the rare disease context—PSbg4.

**Fig. 2:**
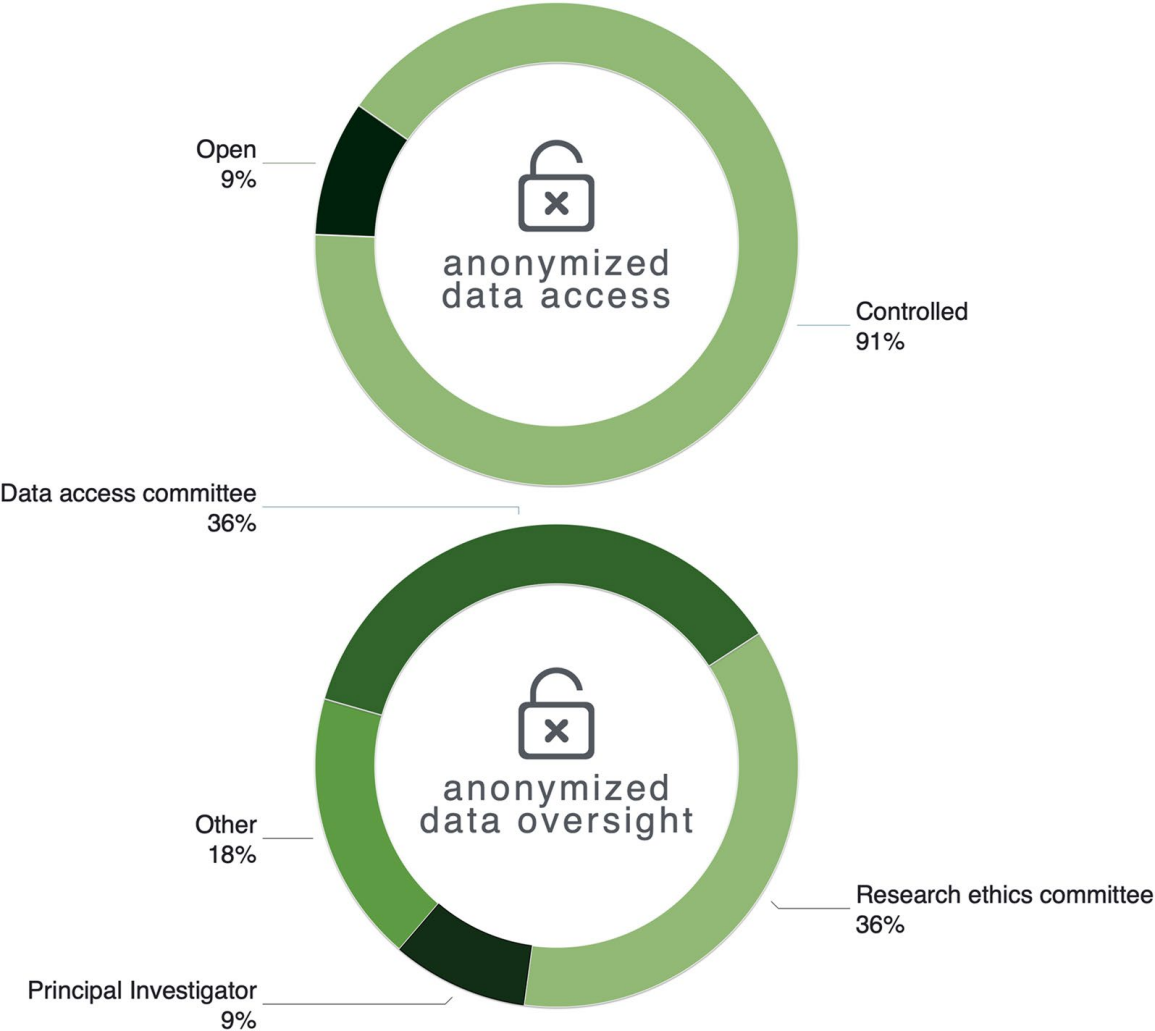
Results from Round 3 survey in response to the question, What is the most appropriate mechanisms of (i) data access and (ii) oversight for the responsible sharing of coded genomic and associated clinical data involving children? N = 10**. **While 12 panelists in total participated in Round 3, only complete survey data from 10 panelists are reported

Consistent with findings from Round 2, the panelists overwhelmingly supported controlled access for coded data (Fig. 2). This majority support is particularly noteworthy given that the panel composition differed most between Rounds 2 and 3. Data that enables “identifiable links to children and their clinical data” (PIcHG) warrants greater access controls because such linkage poses higher informational risks according to 91% of panelists. IRBs and DACs (72%) were identified as having oversight responsibility for such controlled access.

**Fig. 3:**
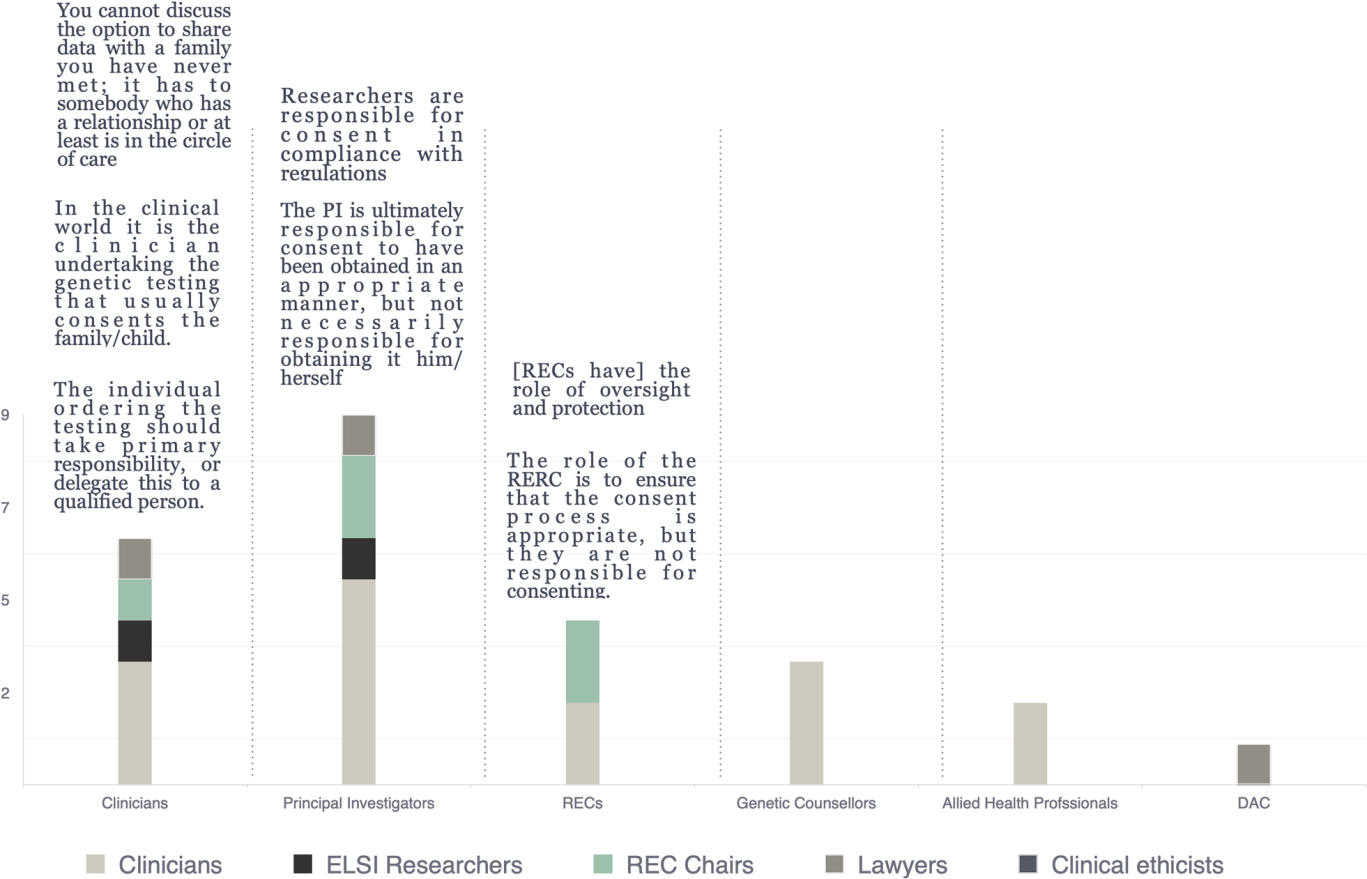
Mixed qualitative and quantitative results from Round 3 survey in response to the question, Who should be responsible for discussing permissions to share genomic and associated clinical data involving children and their families? N = 10**

To clarify the low consensus and polarization for Statement #12, panelists were asked to identify professionals best able to discuss data sharing opportunities with families, as well as the resources needed to support professionals in this process (Fig. 3). A combination of technological, material and human resources emerged from the qualitative content analysis, where electronic consents and interoperable EHR platforms were the most frequently cited resource needs.

Irreconcilable differences on perceived risks associated with, and access to anonymized versus coded data substantiated the decision to suspend the policy Delphi after Round 3. Table 4 presents a ranked list of all validated statements after three rounds according to their combined averages across rating categories.

**Table 4:** Rankings for position statements based on combined average ratings after Rounds 1 and Round 2

#	Position statement (rating category)*		Rank
5	Parental authorization for ongoing, or future unspecified research should include the provision of information related to existing data governance (RI + D)		1
8	The decision to share pediatric genomic and associated clinical data should be supported by an evaluation of realistic risks and benefits (F + C)		2
3	Parents should be informed in a transparent manner how their child’s genomic and associated clinical data will be securely managed and used (RI + C)		3
1	The best interests of children are primary (RI + F)		4
7	Professionals involved in consent processes related to data sharing and data-intensive research have the responsibility to balance potential benefits and risks. A trained designate should be available to discuss these with parents at the time of consent (D + F)		5
9	Duplicative collection of genomic research data involving pediatric patients should be avoided (D + F)		6
13	Incidental (secondary) findings of clinically actionable, validated genomic results should be made available		6
6	Values conveyed by family, legal guardians or primary care givers should be respected when possible (RI + F)		7
2	Children should be listened to, and involved in decision-making processes related to genomic and associated clinical data sharing in developmentally appropriate ways (D + F)		8
4	In a research context, data sharing infrastructures should enable children to withdraw consent to continued sharing of their genomic and associated clinical data when possible upon reaching the age of majority (D + F)		8

Statements #10, #11 and #12 did not meet the criteria for consensus

*RI, relative importance; F, feasibility; C, confidence; D, desirability

## Discussion

Nine original statements from the KIDS Framework, and one new statement were validated in this three-round policy Delphi study with pediatric genomic stakeholders in Canada. The three highest ranked statements corresponded to essential elements in the informed consent process, namely evaluation of realistic risk and benefit (Statement #8), transparency about how data will be managed and used (Statement #3), and the possibility of future unspecified use (Statement #5). Lack of consensus and high polarity with respect to Statements #10–12 centered on the relationship between re-identifiability risks, and how access to anonymized and coded genomic data should be managed. The conceptual difficulty and high comprehension level needed to participate meaningfully in shared decision making were likely reasons Statement #2 ranked low despite wider support in the pediatric bioethics literature for children’s engagement in research decisions. The impossibility of withdrawing research data if already aggregated, and an inability to provide a “menu” of data sharing options to accommodate family values likely explain why Statements #4 and 8 ranked among the lowest relative to other Statements.

Highly polarizing views on the effectiveness with which genomic data could be anonymized prevented consensus on Statements #10 and #11. The same ethics principles were at times used by different stakeholders to simultaneously oppose and support open access sharing of anonymized genomic data. This trend was particularly apparent between clinician-scientists and REB members. Many panelists were conflicted about the acceptable level of informational risks to children, the scope of parental authority to share their child’s genomic data indefinitely, and children’s inability to exercise full decision-making with respect to the access and sharing of their data once reaching the age of majority. Our findings corroborate other studies that report opposition from REB Chairs/members to open access for anonymized genomic data (see for example 26–28). In our study, panelists who served on REB petitioned for stricter user authentications and data access controls than what are currently required in applicable clinical and research data protection regulations.

In contrast, panelists with a clinical background perceived that the risks associated with sharing anonymized data were both minimal and uncontroversial when balanced against the prospect of a molecular diagnosis. Rather, “the significant risk is related to lack of data sharing when it comes to children and slowing the field of research” (Psbg4). It is likely that the overall skepticism expressed in relation to Statements #10 and #11 prevented validation of Statement #12. Implicit in the arguments from panelists with a bioethics background, in particular, was that REBs lack confidence in researchers’ ability to anonymize, or otherwise keep secure children’s genomic data before promoting data sharing opportunities to the extent that Statement #12 endorsed. Despite changes to the panel’s composition between Rounds 2 and 3, the statistical measures of consensus and polarization varied marginally from Round 1. The content analysis furthermore revealed few perceptible differences between early career and senior panelists for Statements that failed to reach consensus.

Enabling open access to identifiable genomic data i.e., without user authentication or registration as a condition for access, runs counter to extant evidence from public opinion studies and data protection regulation. Our study too supports this conclusion. No panelist argued that access to anonymized or coded data should be barred categorically. Panelists disagreed, however, on the appropriate standards that should be applied when determining whether access (open or controlled) to the child’s genomic data (anonymized or coded) advances children’s best interests. Although a clinical best interest standard was considered too high, especially for sharing data in the research setting, clinician-scientists and bioethics researchers were more likely than REB members to factor in the prospect of ancillary benefits to diagnosis for individual patients (Statement #7). Our observation has important implications for clinicians working in genomics-enabled learning health systems whereby clinical and research data sharing are seamlessly merged to support precision diagnosis, care and treatment [23].

Qualitative responses from panelists across all survey rounds suggest that stakeholders appeal to three key considerations for sharing children’s genomic data: (i) the re-identifiability of the data being shared, (ii) that access to the data is appropriate to the risk of re-identifiability, and (iii) adequate oversight of approved uses for the data. Several conclusions may be drawn from this observed relationship. First, recommended data access regimes may be unlikely to obtain stakeholder buy-in until sufficient evidence demonstrates the actual risk of re-identifiability from anonymized genomic data sets, and this evidence can be communicated to guide shared decision making. Second, general skepticism of data anonymization underscored tensions between data sharing practitioners e.g., researchers and clinicians and oversight bodies e.g. IRBs and DACs. Both groups reinforced that IRBs should have additional training in the review and oversight of protocols that involve consenting families on the realistic benefits and risks of genomic data sharing.

Further research is needed to assess this relationship across diverse data sharing contexts, patient populations, jurisdictions and data repositories. In specific, research quantifying the relative informational risks posed to patients and their families when their genomic data are shared is needed if the intractable views surrounding access and governance highlighted in this study are to be resolved. A case-by-case privacy test as part of an institution’s data sharing policy provides one possible approach to better contextualizing the benefits and more proportionately assess the informational risks of an anticipated data sharing activity for children and their families. This test could include, among other measures, an assessment of the family’s privacy disposition [24] relative to the type of data and manner in which the data will be shared i.e. open vs. controlled, in a clinical or research database. Consultation with patients—children if possible and appropriate, as well adult patients—is critical in this regard to ensure an institution’s data sharing policy aligns with the values and priorities of the community it serves.

## Limitations

The results of this study should be interpreted in light of several limitations. The panel size was within the 10–50 range recommended in the policy Delphi literature [17, 18, 25, 26], yet response rates were lower than those reported in similar studies (~ 25%). Consensus among this panel may not therefore suggest how well the KIDS Framework may transfer across other pediatric genomic centers in Canada. This study does not capture the views and perspectives of all interested stakeholders in pediatric genomics. Because the study aimed to validate a framework meant to guide institutional data sharing policy and practice, only institutional stakeholders were recruited to participate on the policy Delphi panel. Input from pediatric patients and their parents is missing from this validation exercise, albeit critical to the future implementation of the KIDS Framework. To reduce survey fatigue and ensure retention among panelists, the study team assigned two, rather than all four possible rating criteria for each statement. Rating assignments were informed by findings from a systematic review of the data sharing literature, but could have narrowed the strength and direction of consensus. While many Delphi studies suffer from high attrition [27, 28], this study achieved higher than usual retention (> 90%). It is possible, however, that inconsistency in the panel’s composition between Rounds 2 and 3 influenced consensus outcomes, particularly involving the most contentious Statements #10–12.

## Conclusions

The ten validated statements provide institutions with empirically supported best practices for sharing genomic and associated clinical data involving children from the perspectives of key stakeholders in pediatric genomics in Canada. The qualitative and quantitative results from this Delphi study identify for institutions and policy makers where contentious areas still lie in the evolving debate on responsible data sharing, namely establishing data access and oversight regimes commensurate with the relative informational risks of sharing sensitive, genomic information. This study is, to the best of our knowledge, first to contribute such empirical policy evidence in Canada. Moreover, this study demonstrates how the policy Delphi is a particularly useful tool for mixed methods researchers that aim to identify key ethical, legal and social issues relevant to genomic data governance, and to capture the normative stalemates preventing broader consensus among interested stakeholders. Future implementation science research in this area is needed that involves more diverse stakeholders across clinical and research data environments, notably pediatric patients, their families, data privacy engineers, genomic data custodians and database managers.

## Supplementary Information

**Additional file 1**. Parameters for consensus, polarity and support adopted from Needham et al 1990 to validate individual position statements in the policy Delphi. Twelve position statements outlined in the Key Implications for Data Sharing Framework were rated on a 4-point Likert scale for relative importance, desirability, feasibility, confidence.** Rating of 1** = very desirable /definitely feasible/very important/ confident;** Rating of 2** = desirable /possibly feasible/ somewhat important/ reliable;** Rating of 3** = undesirable /possibly not feasible/ somewhat unimportant/ risky;** Rating of 4** = very undesirable /definitely not feasible/ very unimportant/ unreliable.

**Additional file 2**. McNemar change tables measuring directions of change in n= 9 panelist ratings between Rounds 1 and 2 for Statements 5–7 and 10–12. Red boxes indicate the number of panelists with a positive degree of change from Round 1 to 2 (i.e. lowered perceived value based on new Likert rating) while yellow boxes indicate the number of panelists with a negative degree of change (i.e. higher perceived value based on new Likert rating).

## Data Availability

The datasets used and/or analysed during the current study are available from the corresponding author on reasonable request.

## Abbreviations

DAC: Data Access Committee
ELSI: Ethical, Legal, Social Issues
KIDS: Key Implications of Data Sharing
IRB: Institutional Review Board
REC: Research Ethics Committee

## Footnotes

1 *infra* iii.
