# Supplementary material for: A policy Delphi study to validate the key implications of data sharing (KIDS) framework for pediatric genomics in Canada
Source: BMC Med Ethics. 2021 Jun 9;22:71. doi: 10.1186/s12910-021-00635-1 (PMC8191056; doi:10.1186/s12910-021-00635-1)
Supplement: Supplementary file 1 — Additional file 1. Parameters for consensus, polarity and support adopted from Needham et al 1990 to validate individual position statements in the policy Delphi. Twelve position statements outlined in the Key Implications for Data Sharing Framework were rated on a 4-point Likert scale for relative importance, desirability, feasibility, confidence. Rating of 1 = very desirable /definitely feasible/very important/ confident; Rating of 2 = desirable /possibly feasible/ somewhat important/ reliable; Rating of 3 = undesirable /possibly not feasible/ somewhat unimportant/ risky; Rating of 4 = very undesirable /definitely not feasible/ very unimportant/ unreliable. [file 12910_2021_635_MOESM1_ESM.docx]

**Supplemental Materials 1**(1)**.** Parameters for consensus, polarity and support adopted from Needham et al 1990 to validate individual position statements in the policy Delphi. Twelve position statements outlined in the Key Implications for Data Sharing Framework were rated on a 4-point Likert scale for relative importance, desirability, feasibility, confidence. **Rating of 1** = very desirable /definitely feasible/very important/ confident; **Rating of 2** = desirable /possibly feasible/ somewhat important/ reliable; **Rating of 3** = undesirable /possibly not feasible/ somewhat unimportant/ risky; **Rating of 4** = very undesirable /definitely not feasible/ very unimportant/ unreliable

**Parameters for consensus:** measures the degree to which the group was able to agree on support.

|  | **Parameter** |
| --- | --- |
| **High** | ﻿70% of ratings in 1 category, or 80% in 2 contiguous categories |
| **Med** | 60% of ratings in 1 category, or 70% in 2 contiguous categories |
| **Low** | 50% of ratings in 1 category, or 60% in 2 contiguous categories |

**Parameters for polarity:** measures whether the group’s ratings were polarized (e.g. 10 0 0 10 is a strongly polarized distribution). Categories include strong, weak, none. Polarity is determined using the variance (VAR.S) of the distribution.

|  | **De Loe 1995** | **Rahimzadeh et al. 2020^[[1]](#footnote-1)^** |
| --- | --- | --- |
| **Strong** | ﻿Higher than 1.5 | ﻿Higher than 1.1 |
| **Weak** | Between 1.2 and 1.5 | Between 0.8976 and 1.1 |
| **None** | Less than 1.2 | Less than 0.8976 |

**Parameters for support**: indicates where the group’s support lay when there was *consensus*. When consensus is ‘none’, support is always ‘ambiguous’. It can also be ‘ambiguous’ when: (1) the level of consensus is ‘low’ and the ratings are divided equally between two categories (e.g. rating distributions of 10 0 0 10, or 10 0 10 0); (2) the ratings are distributed in a pattern such as: 4 10 4 2. In this case, consensus would be considered ‘medium’-but the point of support could be either of ‘SS-WS’ or ‘WS-WO’.

|  | **Support code** |
| --- | --- |
| Strong Support | SS |
| Strong, to weak support | SS-ws |
| Weak support | ws |
| Weak support, to weak opposition | ws-wo |
| Weak opposition | wo |
| Weak, to strong opposition | wo-SO |
| Strong opposition | SO |

1. Thresholds for polarity were transformed to the 80^th^ percentile based on highest variance of the distribution calculated in the Round 1 dataset (1.122) (2). [↑](#footnote-ref-1)
