# Supplementary material for: A policy Delphi study to validate the key implications of data sharing (KIDS) framework for pediatric genomics in Canada
Source: BMC Med Ethics. 2021 Jun 9;22:71. doi: 10.1186/s12910-021-00635-1 (PMC8191056; doi:10.1186/s12910-021-00635-1)
Supplement: Supplementary file 2 — Additional file 2. McNemar change tables measuring directions of change in n= 9 panelist ratings between Rounds 1 and 2 for Statements 5–7 and 10–12. Red boxes indicate the number of panelists with a positive degree of change from Round 1 to 2 (i.e. lowered perceived value based on new Likert rating) while yellow boxes indicate the number of panelists with a negative degree of change (i.e. higher perceived value based on new Likert rating). [file 12910_2021_635_MOESM2_ESM.docx]

**Supplementary Materials 2**. McNemar change tables measuring directions of change in n= 9 panelist ratings between Rounds 1 and 2 for Statements 5-7 and 10-12. Red boxes indicate the number of panelists with a positive degree of change from Round 1 to 2 (i.e. lowered perceived value based on new Likert rating) while yellow boxes indicate the number of panelists with a negative degree of change (i.e. higher perceived value based on new Likert rating).

**Statement 5—**Parental authorization for ongoing, or future unspecified research should include the provision of information related to existing data governance.

| **Relative importance** | | | | | | | | **Desirability** | | | | | |
| --- | --- | --- | --- | --- | --- | --- | --- | --- | --- | --- | --- | --- | --- |
| \|  \| **R2^[[1]](#footnote-1)^** \| \|  \|  \|  \|  \| \| --- \| --- \| --- \| --- \| --- \| --- \| --- \| \| **R1** \| VI \| \| SI \| SU \| VU \|  \| \| VI \| 2 \| \| 3 \|  \|  \| 5 \| \| SI \|  \| \| 2 \|  \|  \| 2 \| \| SU \| 1 \| \|  \| 1 \|  \| 2 \| \| VU \|  \| \|  \|  \|  \| 0 \| \|  \| \| 3 \| 5 \| 1 \| 0 \| 9 \| | | | | \|  \| **R2** \|  \|  \|  \|  \| \| \| --- \| --- \| --- \| --- \| --- \| --- \| --- \| \| **R1** \| VD \| D \| U \| VU \|  \| \| VD \| 4 \|  \|  \|  \| 4 \| \| \| D \| 1 \| 3 \|  \|  \| 4 \| \| \| U \|  \|  \|  \|  \| 0 \| \| \| VU \|  \| 1 \|  \|  \| 1 \| \| \|  \| 5 \| 4 \| 0 \| 0 \| 9 \| \| | | | | |  |  |  |  |  |
| **Statement 6—**Values conveyed by family, legal guardians or primary care  givers should be respected when possible. | | | | | | | | | | | | | |
| **Relative importance** | | | | | | | | **Feasibility** | | | | | |
| \|  \| **R2** \|  \|  \|  \|  \| \| --- \| --- \| --- \| --- \| --- \| --- \| \| **R1** \| VI \| SI \| SU \| VU \|  \| \| VI \| 4 \|  \|  \|  \| 4 \| \| SI \|  \| 3 \|  \|  \| 3 \| \| SU \|  \| 2 \|  \|  \| 2 \| \| VU \|  \|  \|  \|  \| 0 \| \|  \| 4 \| 5 \| 0 \| 0 \| 9 \| | | | \|  \| **R2** \|  \|  \|  \|  \| \| \| --- \| --- \| --- \| --- \| --- \| --- \| --- \| \| **R1** \| DF \| PF \| PNF \| DNF \|  \| \| DF \| 1 \|  \|  \|  \| 1 \| \| PF \|  \| 2 \| 1 \|  \| 3 \| \| PNF \|  \| 1 \| 2 \|  \| 3 \| \| DNF \|  \|  \| 2 \|  \| 2 \| \|  \| 1 \| 3 \| 5 \| 0 \| 9 \| | | | | |  |  |  |  |  |  |
| **Statement 7—**All professionals involved in processes of data sharing and data-intensive research have the responsibility to balance potential benefits and risks and discuss these with parents at the time of consent. | | | | | | |  |  |  |  |  |  |  |
| **Desirability** | | **Feasibility** | | | | |  |  |  |  |  |  |  |
| \|  \| **R2** \|  \|  \|  \|  \| \| --- \| --- \| --- \| --- \| --- \| --- \| \| **R1** \| VD \| D \| U \| VU \|  \| \| VD \| 4 \|  \|  \|  \| 4 \| \| D \| 1 \| 2 \| 1 \|  \| 4 \| \| U \| 1 \|  \|  \|  \| 1 \| \| VU \|  \|  \|  \|  \| 0 \| \|  \| 6 \| 2 \| 1 \| 0 \| 9 \| | | \|  \| **R2** \|  \|  \|  \|  \| \| --- \| --- \| --- \| --- \| --- \| --- \| \| **R1** \| DF \| PF \| PNF \| DNF \|  \| \| DF \| 1 \|  \|  \|  \| 1 \| \| PF \|  \| 4 \|  \|  \| 4 \| \| PNF \| 1 \| 1 \|  \|  \| 2 \| \| DNF \|  \| 1 \|  \| 1 \| 2 \| \|  \| 2 \| 6 \| 0 \| 1 \| 9 \| | | | | |  |  |  |  |  |  |  |
| **Statement 10—**Anonymized pediatric data should be made available via publicly accessible databases. | | | | | | |  |  |  |  |  |  |  |
| **Desirability** | | **Feasibility** | | | | |  |  |  |  |  |  |  |
| \|  \| **R2** \|  \|  \|  \|  \| \| --- \| --- \| --- \| --- \| --- \| --- \| \| **R1** \| VD \| D \| U \| VU \|  \| \| VD \| 3 \| 2 \|  \|  \| 5 \| \| D \| 1 \|  \|  \|  \| 1 \| \| U \|  \|  \| 2 \|  \| 1 \| \| VU \|  \|  \|  \| 1 \| 1 \| \|  \| 4 \| 2 \| 2 \| 1 \| 9 \| | | \|  \| **R2** \|  \|  \|  \|  \| \| --- \| --- \| --- \| --- \| --- \| --- \| \| **R1** \| DF \| PF \| PNF \| DNF \|  \| \| DF \| 3 \|  \|  \|  \| 3 \| \| PF \| 1 \| 2 \|  \|  \| 3 \| \| PNF \| 1 \|  \| 2 \|  \| 3 \| \| DNF \|  \|  \|  \|  \| 0 \| \|  \| 5 \| 2 \| 2 \| 0 \| 9 \| | | | | |  |  |  |  |  |  |  |
| **Statement 11—**Identifiable pediatric genomic and associated clinical data should be coded and made available through a controlled or registered access process. | | | | | | |  |  |  |  |  |  |  |
| **Desirability** | | **Feasibility** | | | | |  |  |  |  |  |  |  |
| \|  \| **R2** \|  \|  \|  \| \|  \| \| --- \| --- \| --- \| --- \| --- \| --- \| --- \| \| **R1** \| VD \| D \| U \| \| VU \|  \| \| VD \| 6 \|  \|  \| \|  \| 6 \| \| D \| 1 \|  \|  \| \|  \| 1 \| \| U \|  \| 1 \|  \| \|  \| 1 \| \| VU \|  \|  \|  \| \| 1 \| 1 \| \|  \| 7 \| 1 \| 0 \| \| 1 \| 9 \| \| | | \|  \| \| **R2** \| \|  \| \|  \| \|  \| \|  \| \| \| --- \| --- \| --- \| --- \| --- \| --- \| --- \| --- \| --- \| --- \| --- \| --- \| \| **R1** \| \| DF \| \| PF \| \| PNF \| \| DNF \| \|  \| \| \| DF \| \| 1 \| \| 3 \| \|  \| \|  \| \| 4 \| \| \| PF \| \| 2 \| \| 2 \| \|  \| \|  \| \| 4 \| \| \| PNF \| \|  \| \|  \| \|  \| \|  \| \| 0 \| \| \| DNF \| \|  \| \|  \| \|  \| \| 1 \| \| 1 \| \| \|  \| \| 3 \| \| 5 \| \| 0 \| \| 1 \| \| 9 \| \| | | | | |  |  |  |  |  |  |  |
| **Statement 12—**Providing children and their parents the opportunity to share genomic and associated clinical data is an obligation of those who generate such data. | | | | | | | | |  |  |  |  |  |
| **Desirability** | | | | | | | **Feasibility** | | | | | |  |
| \|  \| **R2** \|  \|  \|  \|  \| \| --- \| --- \| --- \| --- \| --- \| --- \| \| **R1** \| VD \| D \| U \| VU \|  \| \| VD \| 3 \|  \|  \|  \| 3 \| \| D \| 2 \| 1 \|  \|  \| 3 \| \| U \| 1 \|  \| 1 \|  \| 2 \| \| VU \|  \|  \| 1 \|  \| 1 \| \|  \| 6 \| 1 \| 2 \| 0 \| 9 \| | | | | | | | \|  \| **R2** \| \|  \|  \|  \|  \| \| --- \| --- \| --- \| --- \| --- \| --- \| --- \| \| **R1** \| \| DF \| PF \| PNF \| DNF \|  \| \| DF \| \| 2 \|  \|  \|  \| 2 \| \| PF \| \| 1 \| 1 \|  \|  \| 2 \| \| PNF \| \|  \|  \| 3 \| 1 \| 4 \| \| DNF \| \|  \|  \| 1 \|  \| 1 \| \|  \| 3 \| \| 1 \| 4 \| 1 \| 9 \| | | | | | |  |

1. **R1** = Round 1; **R2**= Round 2; **V**[x] = Very [Important, Desirable, Feasible]; **S**[x] =Somewhat [Important, Feasible]; **D** = Desirable; **U** = Undesirable; P[x] = Possibly [Feasible, Not Feasible]; **D**[x] = Definitely [Feasible, Not Feasible]. [↑](#footnote-ref-1)
